# Associations between hippocampal morphology, diffusion characteristics, and salivary cortisol in older men

**DOI:** 10.1016/j.psyneuen.2017.01.027

**Published:** 2017-04

**Authors:** Simon R. Cox, Maria del Carmen Valdés Hernández, Jaeil Kim, Natalie A. Royle, Sarah E. MacPherson, Karen J. Ferguson, Susana Muñoz Maniega, Devasuda Anblagan, Benjamin S. Aribisala, Mark E. Bastin, Jinah Park, John M. Starr, Ian J. Deary, Alasdair M.J. MacLullich, Joanna M. Wardlaw

**Affiliations:** a Centre for Cognitive Ageing and Cognitive Epidemiology, University of Edinburgh, UK; b Department of Psychology, University of Edinburgh, UK; c Scottish Imaging Network, A Platform for Scientific Excellence (SINAPSE) Collaboration, Edinburgh, UK; d Department of Neuroimaging Sciences, Centre for Clinical Brain Sciences, University of Edinburgh, UK; e School of Computing, Korea Advanced Institute of Science and Technology (KAIST), Daejeon, South Korea; f Edinburgh Delirium Research Group, Geriatric Medicine, University of Edinburgh, UK; g Department of Computer Science, Lagos State University, Lagos, Nigeria

**Keywords:** Cortisol, MRI, Brain, Hippocampus, 3D shape analysis, Ageing

## Abstract

High, unabated glucocorticoid (GC) levels are thought to selectively damage certain tissue types. The hippocampus is thought to be particularly susceptible to such effects, and though findings from animal models and human patients provide some support for this hypothesis, evidence for associations between elevated GCs and lower hippocampal volumes in older age (when GC levels are at greater risk of dysregulation) is inconclusive. To address the possibility that the effects of GCs in non-pathological ageing may be too subtle for gross volumetry to reliably detect, we analyse associations between salivary cortisol (diurnal and reactive measures), hippocampal morphology and diffusion characteristics in 88 males, aged ∼73 years. However, our results provide only weak support for this hypothesis. Though nominally significant peaks in morphology were found in both hippocampi across all salivary cortisol measures (standardised *β* magnitudes < 0.518, *p_uncorrected_* > 0.0000003), associations were both positive and negative, and none survived false discovery rate correction. We found one single significant association (out of 12 comparisons) between a general measure of hippocampal diffusion and reactive cortisol slope (*β* *=* 0.290, *p* = 0.008) which appeared to be driven predominantly by mean diffusivity but did not survive correction for multiple testing. The current data therefore do not clearly support the hypothesis that elevated cortisol levels are associated with subtle variations in hippocampal shape or microstructure in non-pathological older age.

## Highlights

• Elevated cortisol does not appear to be associated with regional variations in hippocampal shape.
• Novel shape morphology analysis applied to study possible effect of cortisol on hippocampus.
• Mean diffusivity in hippocampus is associated with reactive cortisol slope in older men.

## 1 Introduction

Glucocorticoid (GC) production is regulated by the hypothalamic-pituitary-adrenal (HPA) axis, activation of which coincides with a period of need that is normally followed by a return to lower levels. It exhibits a diurnal rhythm (highest just after waking, reducing to a nadir overnight) and can also be increased through reaction to systemic or perceived stress (followed by a swift return to basal levels following resolution of the stressor; Herbert et al., 2006). In humans, older age is related to altered diurnal and reactive profiles (Otte et al., 2005, Heaney et al., 2010), as well as structural brain changes. It has been posited that dysregulation of the HPA axis in old age may lead to chronically elevated GCs (cortisol in humans), which may exert deleterious effects on specific brain structures such as the hippocampus (Sapolsky et al., 1986, Landfield et al., 2007), yet current evidence among older humans is inconclusive. In rodent models, prolonged exposure to repeated instances of restraint stress or exogenous steroids results in reduced synaptic and dendritic complexity in the hippocampus (Cook and Wellman, 2004, McEwen and Gianaros, 2010), a reduction in hippocampal volume and hippocampal cell death (Landfield et al., 1978, Sapolsky et al., 1986, Sapolsky et al., 1990). Human Cushing’s patients (whose primary characteristic is a chronic excess of GCs; Patil et al., 2007) exhibit general cerebral atrophy and hippocampal volume reductions (Starkman et al., 2001, Michaud et al., 2009, Toffanin et al., 2011), though it remains unclear the extent to which hippocampal atrophy is distinct from generalised brain changes.

In non-pathological groups of ageing humans, however, the evidence for significant associations between cortisol levels and hippocampal volume measures is limited. Increasing 24 h cortisol levels over a 5 year period was associated with decreases in hippocampal volume (n = 11; Lupien et al., 1998), yet results from subsequent studies are agnostic. Several studies with larger samples have found no significant relationship between hippocampal volume and cortisol levels, variously measured (Coluccia et al., 2008, Cox et al., 2015b, Gold et al., 2005, Kremen et al., 2010, MacLullich et al., 2005, MacLullich et al., 2006, MacLullich et al., 2012). In contrast, others report either a negative association between hippocampal volume and reactive cortisol (Sindi et al., 2014; n = 32), or with waking cortisol, only when accounting for some, but not other types of interleukin (Sudheimer et al., 2014; n = 28). A larger study reported that evening (but not morning) cortisol levels were associated with smaller hippocampal volume, but that this was not significantly greater than general effects on gray matter (Geerlings et al., 2015; n = 4244). In the same sample as in the current study, we previously found no association between hippocampal volumes and either diurnal (morning or evening) or reactive (start and end of a cognitive stressor) salivary cortisol measures (Cox et al., 2015b). One possible factor underlying the null findings in many of the analyses above could be that effects of cortisol on hippocampal structure may be more subtle than gross hippocampal volumetry can detect, especially among groups of relatively healthy, non-pathological groups.

To investigate this possibility, we analysed two alternative classes of hippocampal measurement which might detect such effects. First, we examine differences in hippocampal morphology to identify any systematic shape differences in particular anatomical subregions subfields (boundaries illustrated in Moretti et al., 2012). The distribution of mineralocorticoid and glucocorticoid receptors (to which GCs bind) varies across hippocampal subfields in humans (e.g. Seckl et al., 1991, López et al., 1998, Medina et al., 2013, Wang et al., 2013) and some rodent studies have identified effects of GC exposure specifically in subregions Cornu Ammonis (CA1) and CA3 only (reviewed in McEwen, 2007); hence it is plausible that humans may also exhibit regional associations with cortisol levels. Among healthy children (n = 17, age range 7–12 years; Wiedenmayer et al., 2006), higher cortisol levels were not associated with hippocampal volume, but were associated with outward deformations in the subiculum on the dorsal surface at the head of the right hippocampus and at the dentate gyrus, and with inward deformations along the lateral aspects of the medial hippocampal segment, though data in older humans is lacking. Second, we provide an exploratory analysis of hippocampal microstructure using indices from diffusion tensor MRI (DT-MRI). This imaging modality exploits the Brownian diffusion of water molecules within the cerebral region of interest. Molecular water diffusion is constrained by microstructural features of brain tissue, such as macromolecules, fibres, and membranes (Jones et al., 2013). Extracted diffusion characteristics include the average magnitude of water diffusion (mean diffusivity; MD) and its directional coherence (fractional anisotropy; FA), and is thought to provide information about local microstructural tissue architecture (Alexander et al., 2007). We note that the hippocampal formation is a complex, heterogeneous structure, which makes direct relationships between diffusion data and specific qualities of hippocampal microstructure difficult to interpret. Nevertheless, grey matter water molecule diffusion may be pertinent to brain and cognitive ageing. Several lines of evidence indicate that hippocampal diffusion may provide a more sensitive biomarker for age-related neurodegenerative disease (reviewed in Weston et al., 2015). In the same overall sample that we examine here, these measures reportedly exhibit stronger cross-sectional associations with cognitive ability in older age than hippocampal volume (Aribisala et al., 2014), suggesting that individual differences in hippocampal microstructure may be an informative biomarker in brain ageing research. Hippocampal mean diffusivity has been identified as potentially sensitive to cortisol levels, though in a smaller group with a wider age range (n = 58, Madsen et al., 2012) in which older participants were not well represented (<10 participants aged over 60 years). In summary, we investigate whether innovative neuroimaging techniques may identify subtler regional associations between cortisol and the hippocampus in non-pathological older age than gross volumetry can detect.

## 2 Methods

### 2.1 Participants

The participants were the same as those described previously (Cox et al., 2015a, Cox et al., 2015b). Briefly, they were drawn from the second wave of the Lothian Birth Cohort 1936 (LBC1936) – a longitudinal ageing study of older community-dwelling adults, all of whom were born in 1936. Initially recruited at age 70 (Wave 1, n = 1091; Deary et al., 2007), they underwent a brain MRI scan about three years later at Wave 2 (age ∼73). From this second wave, male participants (to eliminate any potential confound of gender in this modest sample size; Eisenberger et al., 2007) were invited to participate in a cortisol sub-study based on the following criteria: score ≥24 on the MMSE (Folstein et al., 1975), a score <11 on the depression facet of the Hospital Anxiety and Depression Scale (Snaith, 2003), no diagnosis of neurodegenerative disorders, no history of serious neurological event (as ascertained from the MRI scans by a consultant neuroradiologist; JMW), a complete MRI scan, and not taking any GC, antidepressant or any other prescribed medication (ascertained during a detailed medical interview) that is known to impact HPA axis functioning or hippocampal structure. Forty-eight reported a diagnosis of hypertension, 12 diabetes without complications, 34 hypercholesterolaemia and 35 a history of cardiovascular disease (including angina or myocardial infarction). Of the 118 eligible participants who were invited, 90 (mean age 73.3 years, ranging from 72.4 to 74.3) agreed and gave informed consent.

### 2.2 Cortisol

Circulating levels of cortisol in blood and saliva are well-correlated (Vining et al., 1983, Perogamvros et al., 2010), indicating the utility of saliva samples are acceptable (and less invasive) means of indexing free cortisol levels. Salivary cortisol sampling protocol has previously been described elsewhere (Cox et al., 2015a, Cox et al., 2015b). Briefly, samples were collected using Salivette devices (Sarstedt, Numbrecht, Germany) and stored at −80 °C following collection prior to being shipped to Dresden LabService GmbH, Germany, for assay using a commercial immunoassay kit with chemiluminescence detection (IBL-Hamburg, Hamburg, Germany). Intra-assay variation was 5.1%. Correlation coefficients for regression lines were >0.99, consistent with typical international standards. All measurements are reported in nmol/l. Saliva samples were taken at the start (immediately post-consent) and end (immediately following completion of the final test) of a cognitive testing appointment (which acted as a mild cognitive stressor) and will be referred to as START and END levels. Efforts were made to standardise the appointment to 3 hours post-waking for each participant (M = 3 h 16 mins, SD = 51 mins) which lasted just over 11/2 h (M = 1 h 36 mins, SD = 12mins); further details of appointment format and the cognitive tests used were reported previously (Cox et al., 2015a; Supplementary Material). The experimental neuropsychological tests were novel to the participants and were administered in a novel, unpredictable and uncontrolled environment (Lupien et al., 2007). Participants’ diurnal measures were obtained on a separate weekday. They provided a sample on waking (WAKING) prior to eating or brushing their teeth and at 10pm (EVENING) in their home, prior to attending the appointment. They were asked to record the sampling times. Day length (the lag between WAKING and EVENING) was M = 14 h 10 mins, SD = 1 h, 2 mins. Diurnal slope and reactive slope were computed by subtracting the earlier measure (START or WAKING) from the later measure (END or EVENING). A negative value denotes a decreasing slope.

### 2.3 MRI acquisition

MRI scans were acquired using a 1.5 T GE Signa Horizon HDxt clinical scanner (General Electric, Milwaukee, WI, USA) operating in research mode using a self-shielding gradient set with maximum gradient of 33 mT/m and an 8-channel phased-array head coil. The imaging protocol is fully described elsewhere (Wardlaw et al., 2011). For this particular study, we used coronal T1-weighted volumes acquired with a 3D inversion recovery prepared fast gradient echo sequence (TR/TE/TI = 9.7/3.984/500 ms, flip angle α = 8°, bandwidth 15.63 kHz, voxel size 1 × 1 × 1.3 mm^3^), two axial magnetisation transfer spin echo sequences (TR/TE = 3525/10 ms; one with and other without magnetisation transfer pulse) and 2 mm isotropic single-shot spin echo planar diffusion tensor images (TR/TE = 16500/95.5 ms) acquired in 72 directions. All sequences had field of view in the acquisition plane of 256 × 256 mm^2^.

### 2.4 MRI analysis: structural segmentations

Hippocampal shape models were generated from binary masks obtained semi-automatically from the T1-weighted images. First, approximations of left and right hippocampal segmentations were obtained fully automatically using SUSAN (Smith and Brady, 1997), FLIRT (Jenkinson et al., 2002) and FIRST (Patenaude et al., 2011): tools from the FMRIB Software Library ver 4.1 (Oxford, UK; http://www.fmrib.ox.ac.uk/fsl/) and an age-relevant template (Farrell et al., 2009). The results were visually assessed by a trained image analyst and manually corrected using Analyze™ 10.0 software (Mayo Clinic, Rochester, MN, USA; www.analyzedirect.com) and saved as binary masks. To avoid hippocampal volumes influencing the shape deformation analyses, semi-automated measurements of intracranial volume (ICV; contents within the inner skull table including brain tissue, cerebrospinal fluid, veins and dura) were used for normalisation (Valdés Hernández et al., 2012). Due to movement artefacts, segmentation of the hippocampus was not possible in 2 of the 90 participants, leaving 88 for further analysis.

### 2.5 MRI analysis: shape model analysis

**Fig. 1:**
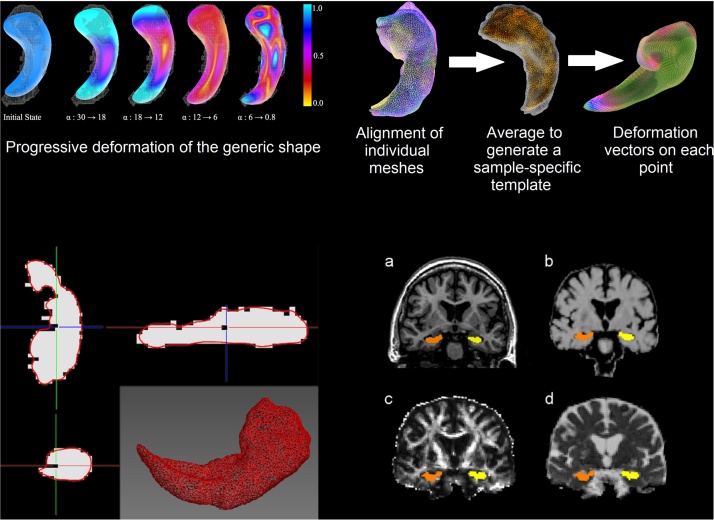
Hippocampal mesh modelling process. Top left: Illustration of the principle of the coarse-to-fine surface deformation of the progressive weighting scheme. The wireframe model represents the target hippocampus’s surface and the solid model represents the hippocampal generic shape model. Colours indicate the distance of the vertices’ translations after surface deformation (mm). Top right: The steps involved in the hippocampal shape analysis. Bottom left: Mid-coronal brain MRI slice of a participant showing the hippocampal binary masks (white) obtained from the T1-weighted scan (a) deformed to fit the anatomical structure as represented in the MTR (b), FA (c) and MD (d) maps. Bottom right: Triangular mesh model of a right hippocampus from a dataset for which the fiducial localisation error was 8.48 mm and the mean distance between the surface mesh model (represented in red) and the binary mask (represented in white) was 0.78 mm. Axial (top left), sagittal (top right) and coronal (bottom left) views showing the fitness of the surface mesh model to the binary mask, illustrating the reasons behind the apparent “unfitness”. (For interpretation of the references to color in this figure legend, the reader is referred to the web version of this article.)

Hippocampal binary masks were input to a novel non-rigid shape modelling framework that uses a progressive model deformation technique built-up on a Laplacian surface representation of multi-level neighbourhood and flexible weighting scheme (Valdés Hernández et al., 2014). The modelling framework is schematically illustrated in Fig. 1. Briefly, the surface of a 3D template or “average” model based on all hippocampi in the sample, as a triangular mesh of 3000 vertices, is non-rigidly deformed in a large-to-small scale to allow recovery of the individual shape characteristics, while minimizing the distortion of point distribution of the general model. This surface deformation is achieved through an iterative process that, at each iteration, decreases a rigidity weight α and the level of neighbourhood in a step-wise way together with the magnitude of the displacement of each vertex. At early iterations, the generic 3D model deforms more largely to reproduce the large shape features of the hippocampus by propagating the external force, guiding each vertex of the general model to the closest image boundary, across the surface. In the iteration process, when the general model is not deformed anymore by the balance between the external force and internal force, the rigidity and the level of neighbourhood are gradually diminished so that the model deforms at smaller regions to reproduce local shape details. To preserve the surface quality and diminish the effect that rough boundaries and noise in the binary masks could pose to the shape analyses, a rotation and scale-invariant transformation that constrains the vertex transformations only to rotation, isotropic scale and translation are applied afterwards. This helps regularising the individual vertex transformations to those of the neighbouring vertices using them as reference. The detailed shape modelling algorithm is described in Kim et al. (2015) and illustrated in Fig. 1. Analysis of shape model quality is described in Supplementary Material.

After the 3000-vertex surface mesh model was fit to each hippocampal binary mask, all meshes were co-registered and scaled using the individuals’ ICV, and an average mesh (i.e. a sample-specific “template”) was generated. Then, this “template” mesh was aligned back to each individual mesh (i.e. one-by-one transformations to “native” space) to calculate the deformation of each vertex for each participant relative to the sample-specific “template”.

### 2.6 MRI analysis: generation of quantitative imaging parameters

From the DTI-MRI we obtained parametric maps of fractional anisotropy (FA) and mean diffusivity (MD) using the FMRIB’s Diffusion Toolbox (http://fsl.fmrib.ox.ac.uk/fsl/fslwiki/FDT). Magnetisation Transfer Ratio (MTR) maps were generated from the signal intensities with and without magnetisation transfer pulse. Hippocampal binary masks were applied to these maps (Fig. 1) using non-linear registration (Modat et al., 2010), as implemented in the TractoR project (Clayden et al., 2011) (http://www.tractor-mri.org.uk/diffusion-processing). Median values of MTR, FA and MD within the hippocampal masks were obtained.

### 2.7 Statistical analysis

As previously reported (Cox et al., 2015a, Cox et al., 2015b), outlying cortisol points (3%) were removed prior to analysis. All of these points were <95th percentile of unpublished diurnal salivary cortisol data from 13,366 humans, collected at Dresden LabService GmbH, where the assays were undertaken (Clemens Kirschbaum, personal communication). Further, there is a poorer correspondence between salivary and serum cortisol levels as concentrations increase due to CBG saturation at higher concentrations resulting in increased free cortisol (Hellhammer et al., 2009). Cortisol measures at EVENING, START and END were log transformed due to skewness. We calculated the association between the deformation vector at each point of the hippocampal triangular meshes and each cortisol measure using general linear modelling in MATLAB R2014a, covarying for age at scan to remove any residual variance present due to Wave 2 duration (∼3 years). We examined left and right hippocampi separately, given the possible hemispheric lateralisation of GC effects (Czéh et al., 2008, Madsen et al., 2012). All p-values (i.e. obtained at each vertex) obtained from evaluating each association (e.g. between the deformation vector at each surface mesh point and a cortisol measure) were corrected using false discovery rate (FDR; Benjamini and Hochberg, 1995) and threshold-free cluster enhancement analysis (Smith and Nichols, 2009, Pernet et al., 2015) for each simultaneous vertex-wise analysis for each hippocampus (3000 tests).

We also explored the association between each cortisol measure and measures of hippocampal microstructure. Given previous evidence that these measures share a high degree of variance (e.g. Penke et al., 2012), we derived a general measure of microstructure (*g*_ms_) for left and right hippocampi. This was achieved by entering magnetisation transfer ratio (MTR), fractional anisotropy (FA) and mean diffusivity (MD) measures into a principal components analysis, and extracting the first unrotated component (supported by the scree slope). This component accounted for 58.3% and 61.4% of the variance (left and right, respectively), with loadings all >0.69 in magnitude. A higher score corresponds to higher FA, higher MTR and lower MD average values. Thus, we would predict that higher cortisol levels and flatter slopes would correlate negatively with *g*_ms_. We then examined associations between *g*_ms_ and each cortisol measure using bivariate linear regression, with age as a covariate.

## 3 Results

### 3.1 Descriptive statistics

Characteristics of participants are shown in Table 1. Participants’ diurnal and reactive cortisol profiles generally showed the expected pattern of the earlier measure > second measure. As previously discussed in more detail (Cox et al., 2015a, Cox et al., 2015b), the majority of participants’ START levels were higher or at similar levels to the level predicted (for their appointment time) by their linearly-modelled diurnal slope in the majority of cases, suggesting that the appointment (acting as a mild cognitive stressor) elicited the desired HPA-axis reactivity. Analysis of the quality of the hippocampal shape modelling process indicated that the surface models accurately reproduced hippocampal shape details (Supplementary Material).

### 3.2 Associations between salivary cortisol and hippocampal morphology

**Fig. 2:**
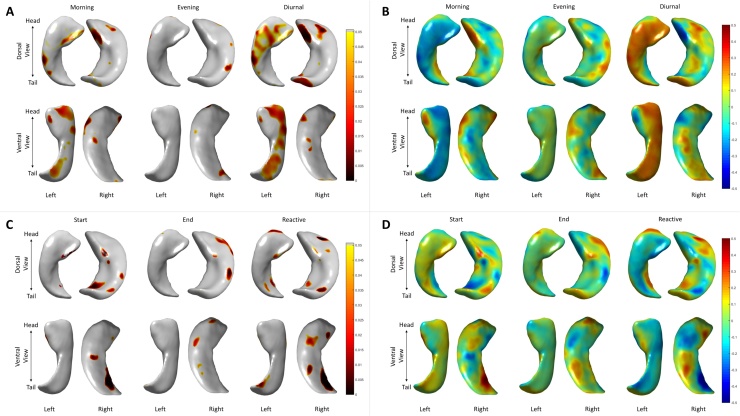
Associations between hippocampal morphology and salivary cortisol. Associations between deformations at each vertex and cortisol are shown for diurnal (A: p-values, B: standardised betas) and reactive (C: p-values, D: standardised betas) salivary cortisol measures.

Regional differences in hippocampal morphology with respect to cortisol levels are shown in Fig. 2. No vertex-wise association with any cortisol measure survived FDR correction for multiple comparisons. However, we provide a brief illustration of the peak effect sizes and uncorrected p-values. There were significant peaks at localised clusters of surface points for all cortisol measures in both left and right hippocampus, and the *β* maps indicated these associations were not exclusively negative (i.e. high cortisol and inward deformation), thus some (non-significant) associations were ostensibly incompatible with the hypothesised deleterious effects of GCs on hippocampal structure. Rather, nominally significant (*p <* 0.05; uncorrected) positive and negative deformations were identified across all cortisol measures – peak positive and negative *β*s are reported in Table 2. Effects showed a tendency to be more highly significant for the right hippocampus. For diurnal slope measures, the most significant relationships were found predominantly on the dorsal surface at the head of the right hippocampus. In contrast, strongest diurnal effects were found in the ventral portion of the right hippocampus.

### 3.3 Associations between salivary cortisol levels and hippocampal diffusion parameters

Table 3 reports the associations between *g*_ms_ and salivary cortisol. These were non-significant apart from the association between reactive slope and right hippocampal *g*_ms_ (*r* *=* 0.290, *p* = 0.008); this effect was not of significantly greater magnitude than for the left hippocampus (*r* *=* 0.206, *p* = 0.064). Further investigation of the individual diffusion parameters indicated that effects of cortisol were only significant for MD measures (Supplementary Table 1). They were strongest (and negative) on MD bilaterally for reactive slope (*r >* −0.299, *p <* 0.01), but also significant (and positive) for morning and start in the left hippocampus (*r* > 0.262, *p <* 0.01).

## 4 Discussion

Following the work of others (Coluccia et al., 2008, Gold et al., 2005, Kremen et al., 2010, MacLullich et al., 2005, MacLullich et al., 2006, MacLullich et al., 2012) and our own previous work with this sample (Cox et al., 2015b) which found no association between gross hippocampal volume and cortisol levels in older age, we hypothesised that advanced MRI techniques may be able to detect the potentially subtler negative effects of cortisol levels on hippocampal structure than gross volumetry might be able to detect. These data provide little support for that hypothesis for either hippocampal shape, or hippocampal microstructure. Whereas some associations at specific loci with specific cortisol measures were highly significant, these were not consistently negatively associated with elevated cortisol (as the central hypothesis would predict; Sapolsky et al., 1986, Landfield et al., 2007), nor did they survive a relatively liberal correction for multiple comparisons.

Nevertheless, there are two interesting results that merit further discussion. First, the pattern of our shape analysis shows a partial overlap with findings from a previous study of hippocampal morphology and cortisol levels in children (Wiedenmayer et al., 2006). They also reported clusters at the subiculum on the dorsal surface at the head of the right hippocampus, and at the dentate gyrus. While glucocorticoid and mineralocorticoid receptors are found throughout hippocampal subfields, the areas reported herein show some coarse correspondence with those areas (CA3 and dentate gyrus) in which both receptor types show the strongest expression in humans (e.g. Seckl et al., 1991, López et al., 1998, Medina et al., 2013, Wang et al., 2013). However, anatomical delineation of hippocampal subfields is complex, and the current surface-based method is unable to evaluate regions that do not lie on the modelled surface. Even with optimal acquisition methods, high field strengths (e.g. Supplementary Fig. 1) and additional approaches (i.e. averaging multiple acquisitions; Adler et al., 2014), the exact correspondence between specific hippocampal receptor subfields and the observed associations reported herein cannot be precisely known. Second, we observed a positive association (non-significant after FDR correction) between a general measure of hippocampal microstructure and reactive slope, which appeared to be predominantly driven by MD. Though the association was in the opposite direction to that which one might expect (here, we found a flatter, or less negative, slope was associated with putatively ‘healthier’ microstructure), we had previously identified a similar association between this same reactive slope and lower (putatively ‘healthier’) MD across white matter tracts of these participants (Cox et al., 2015a, Cox et al., 2015b). In that paper, we cautiously suggested that a positive reactive slope might reflect the appropriate continued activation of the HPA axis, in the face of persistent cognitive challenge. This interpretation may be partly corroborated by the findings for the hippocampus reported herein, which are in the same direction for the same cortisol measure and appear to be driven by the same measure of microstructure (MD). As stated at the outset, despite preliminary evidence that hippocampal diffusion measures may be relevant for brain and cognitive ageing (Madsen et al., 2012, Aribisala et al., 2014, Weston et al., 2015), the microstructural properties that might influence water diffusion in the hippocampus have yet to be clearly elucidated and susceptibility to partial volume effects such as CSF contamination cannot be ruled out, such that these diffusion analyses should be treated with caution.

One of the current study’s limitations is that our sample comprises older adults in relatively good health, reporting only common age-related issues such as hypertension, diabetes, cardiovascular disease and hypercholesterolaemia (for which directional causal relationship with any altered HPA axis functioning is unclear, e.g. Whitworth et al., 2005, Chiodini et al., 2007). Such range restriction hampers our ability to generalise these findings to other samples, where it may be that stronger effects are exhibited in less healthy members of the general population. In addition, they were all male, limiting the applicability of our finding to older females. Thirty-two participants were taking statin medication, which could theoretically affect the biosynthesis of cortisol (Granger et al., 2009). However, substantive reports indicate no effect upon HPA axis functioning nor hippocampal structure (Mol et al., 1989, Doraiswamy et al., 2004). Our study is further limited by the single measurement of salivary cortisol at each time point, when it is recommended that two separate occasions are preferable for diurnal cortisol estimation (Kraemer et al., 2006). Further, compliance with the diurnal (weekday) measures cannot be known because it was unsupervised, though self-report in the current study and previous empirical assessment of compliance rates of this design-type indicate that we could have expected good adherence to the protocol (Kraemer et al., 2006, Almeida et al., 2009).

The present study employed advanced neuroimaging techniques to provide a novel analysis of cortisol levels and markers of subtle hippocampal structural differences in older age. Among its strengths is the narrow age range and well-characterised participant group, removing potential confounds of sex, age, GC medication and depression. The hippocampal masks on which the morphological analysis was based were each visually inspected and manually edited to ensure high quality. The hippocampal modelling method was validated specifically on older individuals experiencing non-pathological ageing, mild cognitive impairment and Alzheimer’s disease (Valdés Hernández et al., 2014). We also used a cohort-specific template to minimise the potential for registration errors and ensured the method could accurately reproduce the hippocampal shape details and correcting for the rough boundaries of the binary masks. Our analyses provide some partial support for associations between salivary cortisol and microstructural properties of the hippocampus, but further work should aim to increase the sample size, examine coupled changes in cortisol levels and neuroimaging measures, and could also apply these methods to other populations in which elevated GC levels are found, such those with mild cognitive impairment and dementia (Popp et al., 2015) and depression (McEwen, 2007, O’Brien et al., 2004).

## Conflicts of interest

None.

## Figures and Tables

**Table 1:** Participant characteristics.

N		88
Age, M (SD), yrs	Mean (SD) years	73.30 (0.37)
Waking	Mean (SD) nmol/l	24 (10.59)
Evening	Mean (SD) nmol/l	3.47 (2.75)
Diurnal Slope	Mean (SD) nmol/l	−20.77 (9.72)
Start	Mean (SD) nmol/l	16.39 (7.77)
End	Mean (SD) nmol/l	12.67 (6.07)
Reactive Slope	Mean (SD) nmol/l	−3.87 (7.19)
Hippocampal FA	Median (IQR)	0.12 (0.016)
Hippocampal MD	Median (IQR) m^2^/s	0.89 (0.064) x10^−6^
Hippocampal MTR	Medianc (IQR) **%**	45.81 (2.045)

*Note.* Descritpive cortisol measures have previously been reported for this group in Cox et al., 2015a, Cox et al., 2015b. Bilateral hippocampal diffusion measures reported (left and right values averaged).

**Table 2:** Peak positive and negative vertex-wise associations between hippocampal morphology and salivary cortisol.

	*Left Hippocampus*				*Right Hippocampus*			
	*Peak +ve β*	*p*	*Peak −ve β*	*p*	*Peak +ve β*	*p*	*Peak −ve β*	*p*
Morning	0.270	**0.0118**	−0.315	**0.00308**	0.308	**0.00383**	−0.272	**0.0109**
Evening^a^	0.234	**0.0337**	−0.291	**0.00794**	0.275	**0.00821**	−0.312	**0.00309**
Diurnal	0.335	**0.00251**	−0.257	**0.0186**	0.311	**0.00391**	−0.348	**0.00141**
Start^a^	0.276	**0.0110**	−0.242	**0.0265**	0.518	**0.0000003**	−0.333	**0.00176**
End^a^	0.232	0.197	−0.227	**0.0325**	0.290	**0.00592**	−0.329	**0.00120**
Reactive	0.284	**0.00855**	−0.300	**0.00578**	0.402	**0.0000941**	−0.459	**0.0000066**

*Note:* Standardised *β* reported. +ve = positive, −ve = negative.

a Log transformed, bold type indicates nominal significance (*p* < 0.05, uncorrected).

**Table 3:** Associations between salivary cortisol and a general factor of hippocampal diffusion, corrected for age.

	*g*_ms_	
	*Left*	*Right*
Morning	−0.203	−0.062
Evening^a^	−0.013	0.027
Diurnal	0.143	0.033
Start^a^	−0.185	−0.137
End^a^	−0.017	0.150
Reactive	0.206	0.290*

*Note:* Standardised *β*s reported.

* *p* = 0.008.

a Log transformed, *g*_ms_ = general factor of hippocampal microstructure. Bold type indicates significance at *p <* 0.05.
