# Supplementary material for: Associations between hippocampal morphology, diffusion characteristics, and salivary cortisol in older men
Source: Psychoneuroendocrinology. 2017 Apr;78:151–8. doi: 10.1016/j.psyneuen.2017.01.027 (PMC5380197; doi:10.1016/j.psyneuen.2017.01.027)
Supplement: Supplementary file 1 [file mmc1.docx]

**Associations between hippocampal morphology, diffusion characteristics, and salivary cortisol in older men.**

**Supplementary Materials**

***Quality Assessment of the Hippocampal Shape Modelling Process***

The quality of the modelling process was evaluated using three metrics: the volumetric similarity index (i.e. Dice coefficient) (Dice, 1945; Zou et al., 2004), and the mean and maximum distances between the individualised surface models and the corresponding binary masks, being the latter known in the technical literature as fiducial localisation error (Fitzpatrick et al., 1998). For calculating the Dice coefficient, the individualised mesh models were converted into binary images. The precision of the modelling method reported in Kim and Valdes-Hernandez et al (2015) is less than half the voxel size. When the Dice coefficient and mean distance suggested this not to be the case, the modelling process was re-done with different values of the rigidity parameter, number of iterations, neighbourhood rings and offsets until a good fit was achieved.

In addition, we assessed our hippocampal shape model by visually evaluating the label assignment and the subfield correspondence on two right hippocampi imaged ex-vivo at 9.4T, using a validated high-resolution atlas (Yushkevich et al. 2009). This right hippocampus atlas is publicly available and can be downloaded from <https://www.nitrc.org/projects/pennhippoatlas/>. The acquisition and labelling process are explained in Yushkevich et al. (2009) and Adler et al (2014).

***Results of the Quality Assessment of the Shape Modelling Process***

Median Dice coefficient values of 0.95 (IQR 0.025) and hippocampal surface-binary mask mean differences of 0.22 mm (IQR 0.18) for left hippocampi and 0.25 mm (IQR 0.31) for the right indicated that the surface models accurately reproduced the hippocampal shape details. The median fiducial localisation error for the left hippocampus was 4.44 mm (IQR 7.58), and for the right hippocampus it was slightly higher 6.87 mm (IQR 7.02). Further investigation revealed that the latter, which measures the maximum distance between the surface model and the binary mask, was high due to rough boundaries on the binary masks effect of the voxelisation (Figure 3) and the presence of small T1-weighted hypointense cavities, referred as “hippocavities” in (Valdés Hernández et al., 2014), some of which have been identified as perivascular spaces and microinfarcts on high field MRI (i.e. 7T) and histology (van Veluw et al., 2013). These are normal features of ageing: some of them may represent a diffuse vascular process with adverse local effects and/or proxies for larger volumes of infarcts or mild or severe diffuse damage.

Results of model quality assessment using publically-available high-field ex-vivo hippocampal data are shown in Figure 1. It illustrates that the modelling process used in our main analyses was able to accurately reproduce the high resolution hippocampal atlas and its labels.


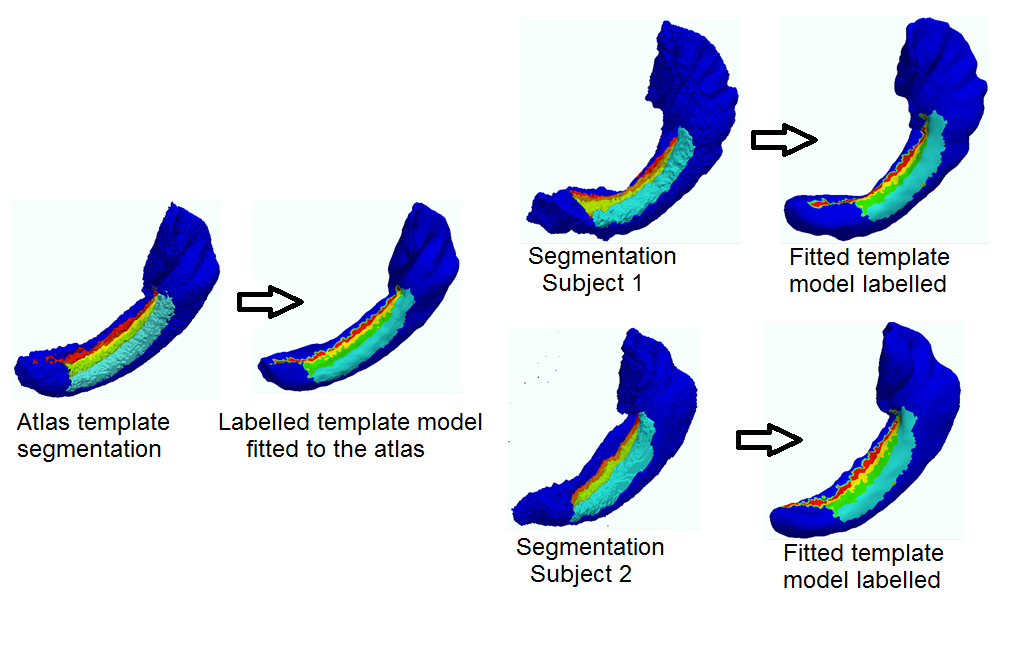


Figure 1. 3D representation of the Segmentation of two post-mortem right hippocampi and the high resolution atlas showing the five following subfields (as per Adler et al. (2014): CA1 (indigo), CA2+CA3 (cyan), CA4/dentate gyrus (green), hilus of the dentate gyrus/subiculum (yellow), stratum radiatum and stratum lacunosum-moleculare/hippocampal sulcus (red), and fitted template model labelled.

*Table S1.* Bivariate correlations between salivary cortisol and individual measures of hippocampal microstructure.

|  | **MTR** | | **FA** | | **MD** | |
| --- | --- | --- | --- | --- | --- | --- |
|  | **L** | **R** | **L** | **R** | **L** | **R** |
| **Morning** | -.102 | .002 | -.038 | -.087 | .**262*** | .093 |
| **Evening** | -.103 | -.172 | .165 | .141 | .113 | .022 |
| **Diurnal** | -.028 | -.104 | .087 | .110 | -.193 | -.064 |
| **Start** | -.082 | -.114 | -.129 | -.089 | **.266*** | .186 |
| **End** | -.081 | .052 | -.055 | .056 | -.029 | -.201 |
| **Reactive** | .061 | .213 | .134 | .160 | **-.299**** | **-.342**** |

*Note.* * *p* <.05, ** *p* < .01. Pearson’s *r* reported; bold type indicates significant association.

**References**

Adler DH, Pluta J, Kadivar S, Craige C, Gee JC, Avants BB, Yushkevich PA, 2014. Histology-derived volumetric annotation of the human hippocampal subfields in postmortem MRI. Neuroimage 84, 505-523.

Dice LR. 1945. Measures of the amount of ecologic association between species. Ecology 26:297-302.

Fitzpatrick JM, Hill DLG, Shyr Y, West J, Studholme C, Maurer CR. 1998. Visual assessment of the accuracy of retrospective registration of MR and CT images of the brain. IEEE Trans Med Imag 17:571–585.

Kim J, Valdes-Hernandez MC, Royle NA, Park J. 2015. Hippocampal Shape Modeling Based on a Progressive Template Surface Deformation and its Verification. IEEE Trans Med Imag 34:1242-1261.

Valdés Hernández MC, Kim J, Whitteford I, Qiu X, Wardlaw JM, Park J. 2014. Automatic hippocampal multimodal assessment for studies of stroke and small vessel disease. Proceedings of the Medical Image Understanding and Analysis (MIUA) 2014 Conference, 9-11 July 2014, London, pp. 33-38. <http://www.staff.city.ac.uk/~sbbh653/MIUA2014Proceedings.pdf>

Van Veluw SJ, Wisse LEM, Kuijf HJ, Spliet WG, Hendriske J, Luijten PR, Geerlings MI, Biessels GJ. 2013. Hippocampal T2 hyperintensities on 7 Tesla MRI. Neuroimage Clin 3:196-201.

Yushkevich, PA, Avants BB, Pluta J, Das S, Minkoff D, Mechanic-Hamilton D, Glynn S. et al. 2009. A High-Resolution Computational Atlas of the Human Hippocampus from Postmortem Magnetic Resonance Imaging at 9.4 Tesla. Neuroimage 44:385-398.

Zhou KH, Warfield SK, Bharatha A, Tempany CMC, Kaus MR, Haker SJ, Wells III WM, Jolesz FA, Kikinis R. 2004. Statistical validation of image segmentation quality based on a spatial overlap index. Acad Radiol 11:178-189.
